# Georgian national palliative care strategic plan — achievements, challenges and perspectives in implementation palliative care in Georgia

**DOI:** 10.1186/1878-5085-5-S1-A106

**Published:** 2014-02-11

**Authors:** T Rukhadze, T Kezeli, T Lekashvili, D Kordzaia

**Affiliations:** 1 Georgian National Academy for Palliative Care – Practical, Educational and Research Resource Centre, Georgian National Association for Palliative Care Faculty of Medicine of Iv. Javakhishvili Tbilisi State University, Georgia

## The aim of the research

is to identify the current status of palliative care as a sum of the legal, clinical, education and research issues, outline the challenges and the ways of its further development.

## Legal issues

• In 2007 Parliament of Georgia has approved the amendments in four different Laws of Georgia supporting development of Palliative Care (PC) System and harmonizing the opioids availability, accessibility and prescription with International standards;

• According to the “Georgian Law of Health Care” the PC is non-separated part of Continuing Medical Aid (together with prevention, treatment and rehabilitation);

• In 2008 the PC guideline (instruction) was approved by Ministry of Health;

• PC is recognized as a subspecialty of Oncology, Critical Care, Internal Medicine and Surgery;

• The Georgian National Association for PC and the Office of coordinator of PC National Program (at the Health Care Committee of Parliament of Georgia) established;

• The National Plan of PC for next five years (2011-2015) approved by Parliament of Georgia.

## Clinical practice

• Home-based PC as Well as in-patients PC was initiated in the Capital of Georgia, Tbilisi since 2004;

• Currently the several PC programs funded by Governmental Budget are implemented in Tbilisi and five regions of Georgia;

• The PC programs for AIDS patients funded by Global Found are running In Tbilisi and three different regions of Country;

## Education

• From 2006 the PC was incorporated in the educational curricula of three Medical Universities as a mandatory (Tbilisi State University) or voluntary (Tbilisi State Medical University, Batumi State University) courses.

• Several editions of Georgian PC Handbooks for medical and nursing students as well as healthcare professionals working in cancer and/or pediatric PC – are issued.

## Research

• During the last years the several research projects in the field of PC were funded by “Open Society - Georgia Foundation” and Georgian National Science Foundation;

• Several papers and abstracts are published in International PC Journals and Congress’ Abstract books;

• PhD course in PC was implemented since 2012 and already 3 PhD students working in this direction.

## Challenges

• The main challenges on the way of PC development are:

a) Lack of adequate information among the society (customers) as well as potential stakeholders and decision-makers;

b) Lack of knowledge and competences among healthcare professionals;

c) Lack of finances exacerbated by world economic crisis.

## Prospective

• Increase in the educational activities and informational campanies in cooperation of international organizations and experts,

• to involve more donors and sponsors and

• to elaborate, implement and incorporate in health care system quality eccuarence tools and standarts in PC seem to be the real ways of further development of PC in Georgia.

